# The Diterpene Isopimaric Acid Modulates the Phytohormone Pathway to Promote *Oryza sativa* L. Rice Seedling Growth

**DOI:** 10.3390/cimb46090580

**Published:** 2024-09-02

**Authors:** Jiaqi Huang, Juan Hua, Luying Peng, Liping Bai, Shihong Luo

**Affiliations:** Engineering Research Center of Protection and Utilization of Plant Resources, College of Bioscience and Biotechnology, Shenyang Agricultural University, Shenyang 110866, China; jiaqihuang@stu.syau.edu.cn (J.H.); huajuan@syau.edu.cn (J.H.);

**Keywords:** growth and defense, isopimaric acid, phytohormone, plant growth regulator, relevance analysis

## Abstract

Many plant secondary metabolites are active and important in the regulation of plant growth. Certain plant-derived diterpenes are known to promote plant growth, but the pathways by which this promotion occurs are still unknown. Activity screening revealed that the plant-derived diterpene isopimaric acid exhibits growth-promoting activity in rice (*Oryza sativa* L.) seedlings. Furthermore, 25 μg/mL of isopimaric acid promoted the growth of 15 self-incompatible associated populations from different rice lineages to different extents. Quantitative analyses revealed a significant decrease in the concentration of the defense-related phytohormone abscisic acid (ABA) following treatment with isopimaric acid. Correlation analysis of the phytohormone concentrations with growth characteristics revealed that the length of seedling shoots was significantly negatively correlated with concentrations of 3-indole-butyric acid (IBA). Moreover, the total root weight was not only negatively correlated with ABA concentrations but also negatively correlated with concentrations of isopentenyl adenine (iP). These data suggest that isopimaric acid is able to influence the phytohormone pathway to balance energy allocation between growth and defense in rice seedlings and also alter the correlation between the concentrations of phytohormones and traits such as shoot and root length and weight. We provide a theoretical basis for the development and utilization of isopimaric acid as a plant growth regulator for rice.

## 1. Introduction

Plant-specialized metabolites play important roles in the regulation of plant growth and include phenolics, terpenoids, and alkaloids [1,2]. Endogenous phenolics are known to participate in indole acetic acid (IAA) synthesis or catabolism, affecting IAA concentrations in plants and playing a regulatory role in the process of plant stem elongation [3]. Some exogenous phenolics also have a function in regulating plant growth. Five plant-derived phenolic compounds isolated from the roots of *Panax quinquefolius* L., salicylic acid, cinnamic acid, coumaric acid, vanillic acid, and butyric acid, all significantly inhibited rhizome growth in *P. quinquefolius* seedlings [4]. Some of these phenolic acids have been found to promote the formation of stress granules (SGs) by binding to the receptor protein RBP47B. SGs ultimately inhibit plant growth through the inhibition of translation [5]. Terpenoid-specialized metabolites of plant origin have a similarly wide range of chemical diversity and biological activities. For example, both exogenous and endogenous plant sources of abscisic acid and monocotyledonolactone regulate plant growth processes by promoting leaf abscission and root growth [6]. The triterpenoids marneral, thalianol T1, arabidiol A1, tirucalladienol Tr1, and baruol B1 secreted from *Arabidopsis* roots regulate growth by influencing the inter-root microbial community formed by 19 species of bacteria, including *Ascomycetes*, *Actinobacteria*, and *Thickettsia*, which are involved in plant growth and acclimatization to environmental changes [7]. Furthermore, the diterpenoid polyalthic acid isolated from the white-haired torch flower (*Colquhounia vestita* Wall.) can help *Arabidopsis thaliana* withstand UV and cold stress by increasing seedling biomass [8].

Phytohormones are key substances that regulate plant growth and development, helping plants to sense and respond to external stresses and optimize their growth under changing environmental conditions [9,10]. The phytohormone pathways include those that are growth-related, based on IAA-related phytohormones, cytokinins, and gibberellins, as well as defense-related phytohormones based on abscisic acid, salicylic acid, and JA. When external environmental changes are caused by biotic stresses, including herbivore feeding, and abiotic stresses such as drought, plants activate hormonal pathways to minimize the effects of environmental changes [11]. Certain plant growth regulators also work through phytohormones, for example, oleoresinosterol has a significant promotional effect on plant growth [12,13]. The addition of oleoresinosterol to tomato (*Solanum lycopersicum* L.) pericarp disc tissue promotes fruit ripening, which was associated with a decrease in the chlorophyll and abscisic acid concentration and a significant increase in the lycopene and carbohydrate concentration in the pericarp, mediated via the ethylene pathway [14].

Rice (*Oryza sativa* L.) has a long history of both cultivation and consumption. It is one of the world’s three major food crops and a source of staple food for about 4 billion people [15]. Understanding the mechanisms of rice growth and development is critical in improving rice quality and yield, as well as enhancing the adaptability of the rice plant to a changing environment [16]. Studies investigating rice height-related genes have demonstrated that shoot changes during growth are regulated by a variety of hormones, including gibberellins (GAs), growth hormones (IAAs), oleoresinolides (BRs), and solanocarpine lactones (SLs), and others [17].

Plant growth regulators stabilize and increase crop yield, improving quality and enhancing crop resistance to herbivores and environmental changes, by regulating the plant growth process [18]. Commercialized plant growth regulators such as paclobutrazol, meperidine, chlormequat chloride, and thiabendazole have been used in crops and have gradually become an important factor in agricultural production [19]. However, there are problems associated with their use, as few varieties of plant growth regulators are known, and their functional mechanisms are unclear, so the search for new plant growth regulators, as well as investigation into the mechanisms of action of existing plant growth regulators, is a major current research hotspot.

In this study, we used growth activity screening and found that isopimaric acid had significant activity in promoting the growth of rice seedlings. Based on this, we interpreted the mechanism of action by which isopimaric acid is able to promote rice (*Oryza sativa* L.) growth, using further activity screening combined with hormonal pathway analysis and correlation analysis.

## 2. Materials and Methods

### 2.1. Plant Materials and Culture Conditions

Nipponbare (*Oryza sativa* L.) rice seeds were obtained from 14 autogamous populations from different rice lineages, including B148_B148, CJCHC4, CJCx59, CJCs214, JRC022, JRC014, JRC042, JRC053, NG28, NG31, NG34, NG45, WRs260, and SUP049. Seeds were cultured in a greenhouse with a photoperiod of 16/8 h (day/night), relative humidity of 65–70%, a light intensity of 1200 lx, and a temperature of 25 ± 2/22 ± 2 °C (day/night).

### 2.2. Extraction of Isopimaric Acid

About 20 kg of North American incense cedar was subjected three times in succession to methanol extraction and combined to obtain a 715 g sample. The extracts were fractionated in silica gel columns and eluted with dichloromethane–acetone (*v*/*v*, 1:0, 9:1, 4:1, 1:1, and 0:1) to yield six fractions (fractions A–F). Fraction A was subjected to ODS gel column chromatography and eluted with a methanol/water gradient (80, 90, 95, and 100%) to yield subfractions (B1–B5). Subfraction B1 was chromatographed on a sephadex LH-20 column (with acetone as the eluent) and was finally purified with semi-preparative HPLC (80% methanol in water; 3 mL/min; Waters X-bridge BEH C_18_, 5 μm, 10 × 250 mm) to obtain the compound isopimaric acid. The structure of the isopimaric acid was determined by means of 1D NMR (Bruker, AVANCE II 600 MHz, Karlsruhe, Germany) [20].

### 2.3. Preparation of Reagent Solutions

The preparation of the isopimaric acid solution: 25, 12.5, 6.25, or 3.125 mg of isopimaric acid dissolved in 20 μL of DMSO (≤5%) was added to a 2 mL centrifuge tube. A small amount of Tween-20 was added to help solubilization, and the tube was put into an ultrasonic cleaner for 15 min until the isopimaric acid was completely dissolved. Rice hydroponics solution was then added to a final concentration of 50, 25, 12.5, 6.25, or 0 μg/mL.

The preparation of the gibberellin (GA_3_) solution: 25, 12.5, 6.25, or 3.125 mg of GA_3_ dissolved in deionized water was added to a 2 mL centrifuge tube, and the tube was placed into an ultrasonic cleaner for 15 min until the GA_3_ was completely dissolved. Rice hydroponics solution was then added to a final concentration of 50, 25, 12.5, or 6.25 μg/mL. 

### 2.4. Growth of Rice with Different Concentrations of Isopimaric and Gibberellic Acids

After seed germination, rice roots were evenly spread into a 72-well 2 mL centrifuge tube incubator with a mesh screen. Different concentrations of isopimaric acid and gibberellic acid (GA_3_) were then added together with water, and the incubator was placed in the incubation room with a temperature of 25 ± 2/22 ± 2 °C (day/night), a light exposure of 16 h, a darkness exposure of 8 h, and a light intensity of 1200 lx. Each group of treatments was replicated three times and labeled. 

### 2.5. Analysis of Seedling Growth

The phenotypes of the rice seedlings subjected to the different treatments were recorded. The lengths of the aboveground parts (shoot length) and the lengths of the main roots (root length) were measured using a ruler, and the weight of the aboveground parts (shoot weight) and the total root weight (root weight) were obtained using a 10,000-millionths of a kilogram balance. The “Percentage growth rate” function in Excel (Microsoft^®^ Excel^®^ 2021MSO) was used to calculate the promotion of shoot length and shoot weight.

### 2.6. Extraction and Quantitative Analysis of Phytohormones

A 0.5 g sample of the roots or shoots of each rice seeding was mashed with a glass rod. The aboveground and belowground parts were each then separately subjected twice to methanol extraction, and the extracts from the above- and belowground parts were pooled separately. The extracts were then centrifuged. The supernatants were passed through an HLB column, and the resulting eluents were passed through an MCX column. The obtained samples were evaporated to dryness and re-dissolved in 500 μL of chromatographic methanol and then used for UPLC-MS/MS analysis (Shimadzu UPLC-MS/MS 8050, Kyoto, Japan). The analytical conditions were as follows: mobile phase A was 0.1% formic acid in water, mobile phase B was acetonitrile, and the gradient elution procedure was 0–12 min, 5–95% B; 12–14 min, 95–95% B; 14–15 min, 95–5% B; 15–18 min, 5–5% B; 15–18 min, 5–5% B; 15–15 min, 5–5% B; and 18 min, 5–5% B, at a flow rate of 0.2 mL/min. MS detection was performed in MRM mode, and the compound-specific MRM parameters for all tested compounds are shown in Supplementary Table S2.

### 2.7. Phytohormone Standard Curve

A concentration gradient (5, 2, 1, 0.5, 0.2, 0.1, 0.05, 0.02, and 0.01 μg/mL) was prepared for each of the 13 phytohormone standards in chromatographic-grade methanol. Standard samples were then analyzed using UPLC-MS/MS, and the standard curves were plotted based on the linear relationship between the peak area and the concentration of the compounds. These calibration equations were then used to analyze the compounds in the rice samples. The calibration equations of the tested compounds are given in Supplementary Table S2.

### 2.8. Statistical Analyses

Excel was used to record all the data, and the data were expressed as means ± standard deviation (means ± SD). When the data were normally distributed, independent-samples *t*-tests were used to analyze and compare the data differences between two groups using IBM SPSS Statistics 24.0. One-way ANOVA was used to analyze and compare differences between treatments using the LSD test, with *p* < 0.05 considered to be statistically significant. The results were plotted and visualized in GraphPad Prism 8. Increases and decreases in growth rate were calculated using the standard functions in Excel (Microsoft^®^ Excel^®^ 2021MSO). Phytohormone concentrations were plotted as a heat map against growth traits such as length and weight using R (www.r-project.org), and Pearson correlation analyses were then conducted.

## 3. Results

### 3.1. Isopimaric Acid Promotes Rice Seedling Growth

**Figure 1:**
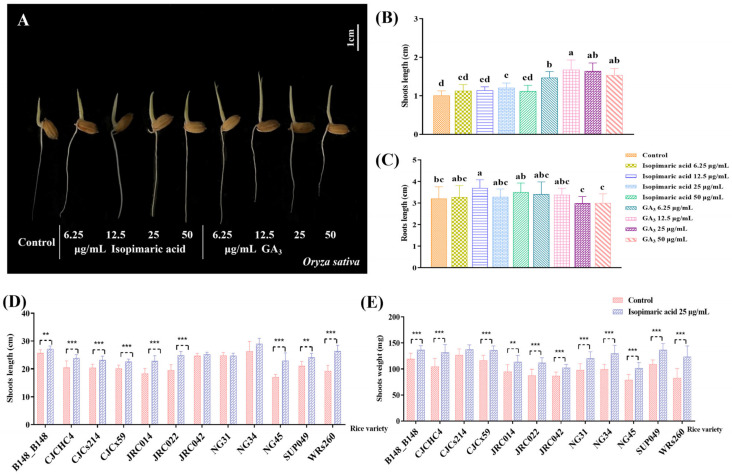
The shoot lengths of 14 rice seedling varieties that received isopimaric acid. (**A**) Growth of Japanese Nipponbare rice seedlings treated with different concentrations of gibberellin and isopimaric acid solution treatment for 3 d. (**B**) Effect of different concentrations of gibberellin and isopimaric acid solution on the length of the shoots of Nipponbare rice seedlings following treatment for 3 d. (**C**) Effect of different concentrations of gibberellin and isopimaric acid solution on the length of the roots of Nipponbare rice seedlings following treatment for 3 d. (**D**) Above-ground lengths of rice seedlings treated with 25 μg/mL of isopimaric acid. (**E**) Effect of isopimaric acid at 25 μg/mL on the root weights of Nipponbare rice seedlings. Differences in data between treatments were compared using one-way ANOVA analysed using the LSD test, and differences between data were considered statistically significant at *p* < 0.05, which was indicated by a, b, c, d, and e. Mean differences between every two groups were compared using independent-samples *t*-tests, with *p* < 0.01 indicated by “**”, and *p* < 0.001 indicated by “***”. *** represents an extremely significant difference between the two sets of data.

The growth activity screening revealed that isopimaric acid promoted the growth of rice seedlings. Isopimaric acid treatment at a concentration of 25 μg/mL for 3 days had the most significant growth-promoting effect compared with gibberellin (Figure 1A–C), and increases in shoot length (Figure 1D) and shoot weight (Figure 1E) were seen in several rice seedlings. After treatment with isopimaric acid at a concentration of 25 μg/mL, seedlings from five rice lines demonstrated significant shoot length and weight promotion (*p* < 0.001): CJCHC4, CJCx59, JRC022, NG45, and WRs260. In seedlings of three rice lines (CJCs214, JRC014, and WRs260), shoot length was significantly promoted (*p* < 0.001), but shoot weight was not. Of these, seedlings of the WRs260 line had an overall highest shoot length promotion of 36.48 ± 1.66%. In addition, rice seedlings from five rice lines showed significant promotion of shoot weight, but not shoot length (*p* < 0.001), following treatment with isopimaric acid at 25 μg/mL, including B148_B148, JRC042, NG31, NG34, and SUP049, with the greatest shoot weight increase seen in WRs260, with an increase of 49.00 ± 26.68%.

### 3.2. Isopimaric Acid Activates the Endogenous Phytohormone Pathway Affecting Rice Growth

#### 3.2.1. Quantification of IAA-Related Phytohormones in Seedlings Showing Growth Promotion

**Figure 2:**
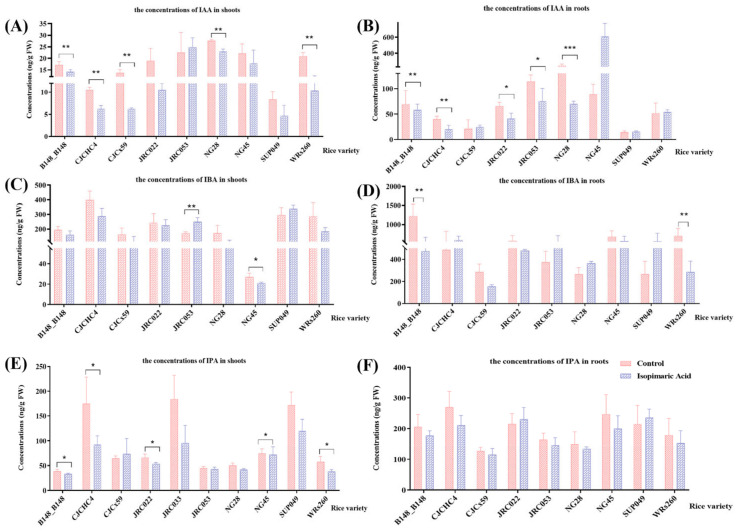
The growth phytohormone-related of 14 rice seedling varieties received isopimaric acid. (**A**,**C**,**E**): IAA, IBA, and IPA concentrations in the shoots of rice seedlings treated with 25 μg/mL of isopimaric acid. (**B**,**D**,**F**): IAA, IBA, and IPA concentrations in the roots of rice seedlings treated with 25 μg/mL of isopimaric acid. Mean differences between the two groups were compared using independent-samples *t*-tests, with *p* < 0.05 indicated by “*”, *p* < 0.01 indicated by “**”, and *p* < 0.001 indicated by “***”. *** represents an extremely significant difference between the means of the two sets of data.

IAA-related phytohormones were quantitatively analyzed in the shoots and roots following treatment with isopimaric acid. The phytohormones tested included IAA, 3-indole-butyric acid (IBA), and 3-indolepropionic acid (IPA) (Figure 2). Compared with the control group, after 25 μg/mL isopimaric acid treatment, the concentrations of IAA, IPA, and IBA all showed a decreasing trend in both the shoots and roots, with the values ranging from 4.72 to 338.74 ng/g FW and from 15.41 to 589.63 ng/g FW, respectively. The concentrations of IAA were significantly reduced in the shoots of seedlings from five rice lines including B148_B148, CJCHC4, CJCx59, and WRs260, with CJCx59 showing the greatest rate decrease in the shoots, at 54.44 ± 0.94%. There were no significant differences in the values of the growth phytohormones in the seedlings from the other six rice lines. In addition, the concentrations of the IAA were significantly increased in the shoots of seedlings of several rice varieties following isopimaric acid treatment, of which the increase in IBA concentrations in the shoots of seedlings of the JRC053 line was the highest, at 45.34 ± 18.80%.

#### 3.2.2. Quantification of Cytokinin Concentrations in Rice Seedlings Following Isopimaric Acid Treatment

**Figure 3:**
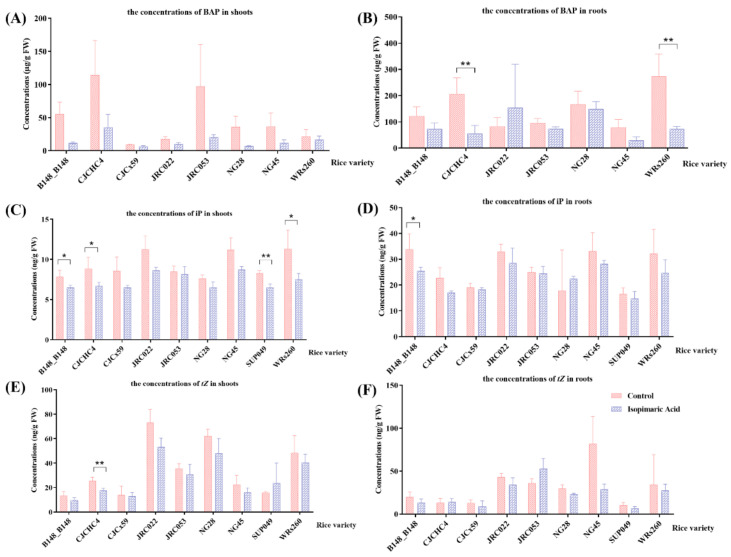
The cytokinin-related phytohormones in 14 rice seedling varieties that received isopimaric acid. (**A**,**C**,**E**): BAP, iP, and *tZ* concentrations in the shoots of rice seedlings treated with 25 μg/mL of isopimaric acid. (**B**,**D**,**F**): BAP, iP, and *tZ* concentrations in the roots of rice seedlings treated with 25 μg/mL of isopimaric acid. Mean differences between every two groups were compared using independent-samples *t*-tests, with *p* < 0.05 indicated by “*” and *p* < 0.01 indicated by “**”.

After isopimaric acid treatment, CK-related phytohormones, including 6-benzyl adenine (BAP), isopentenyl adenine (iP), and *trans*-zearalenin (*tZ*), were quantitatively analyzed in rice seedling shoots (Figure 3). Compared with the control group, the concentrations of BAP, iP, and *tZ* in the roots and shoots of the group treated with isopimaric acid showed a decreasing trend. The concentrations of iP in SUP049 and of *tZ* in CJCHC4 were significantly reduced in the shoots, with the latter having the greatest reduction, 31.00 ± 2.49%. The concentrations of BAP in the roots of two varieties of rice, CJCHC4 and WRs260, were significantly reduced. 

#### 3.2.3. Quantitative Analysis of Gibberellin-Related (GA-Related) Phytohormones in Rice Seedlings Following Treatment with Isopimaric Acid

**Figure 4:**
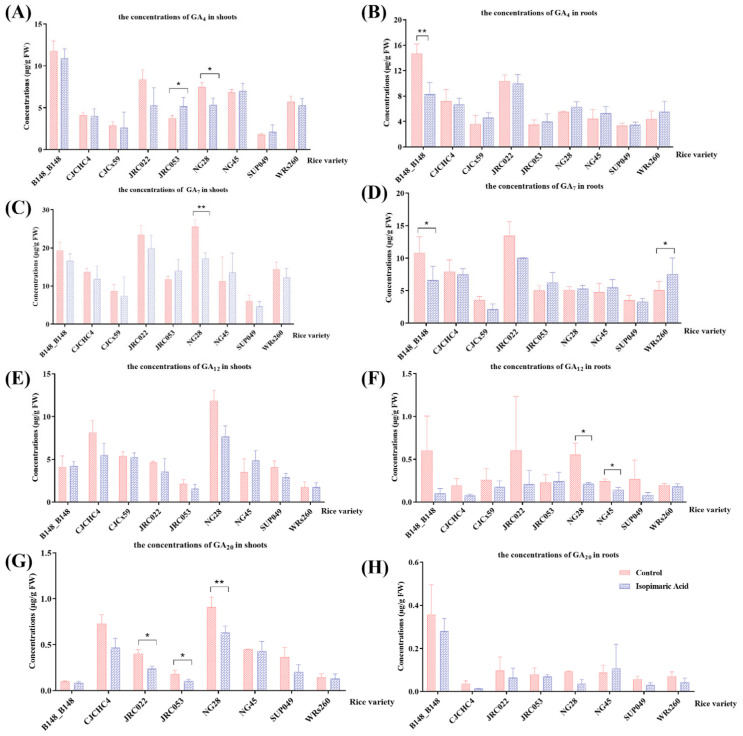
The gibberellin-related phytohormones of 14 rice seedling varieties that received isopimaric acid. (**A**,**C**,**E**,**G**): GA_4_, GA_7_, GA_12_, and GA_20_ concentrations in the shoots of rice seedlings treated with 25 μg/mL of isopimaric acid. (**B**,**D**,**F**,**H**): GA_4_, GA_7_, GA_12_, and GA_20_ concentrations in the roots of rice seedlings treated with 25 μg/mL of isopimaric acid. Mean differences between two groups were compared using independent-samples *t*-tests, with *p* < 0.05 indicated by “*” and *p* < 0.01 indicated by “**”.

Following treatment with 25 μg/mL of isopimaric acid, GA-related phytohormones, including gibberellic acid 4 (GA_4_), gibberellic acid 7 (GA_7_), gibberellic acid 9 (GA_9_), gibberellic acid 12 (GA_12_), and gibberellic acid 20 (GA_20_), were qualitatively analyzed in the shoots and roots of rice seedlings (Figure 4). Compared with the control group, following treatment with 25 μg/mL of isopimaric acid, the concentrations of GA_4_, GA_7_, GA_9_, GA_12_, and GA_20_ decreased in the shoots and roots of most tested rice varieties, with the values ranging from 0.083 to 19.91 μg/g FW and from 0.013 to 10.06 μg/g FW, respectively. Among them, the concentrations of GA_7_ and GA_20_ were significantly reduced (ng/g) in the shoots of NG28 rice seedlings, and the concentration of GA_7_ in the shoots of NG28 was reduced by 32.53 ± 2.65%, with no significant differences seen in the values of gibberellin phytohormones in the rice seedlings of the other eight varieties. 

#### 3.2.4. Quantification of Defense-Related Phytohormone Concentrations in Rice Seedlings Following Treatment with Isopimaric Acid

**Figure 5:**
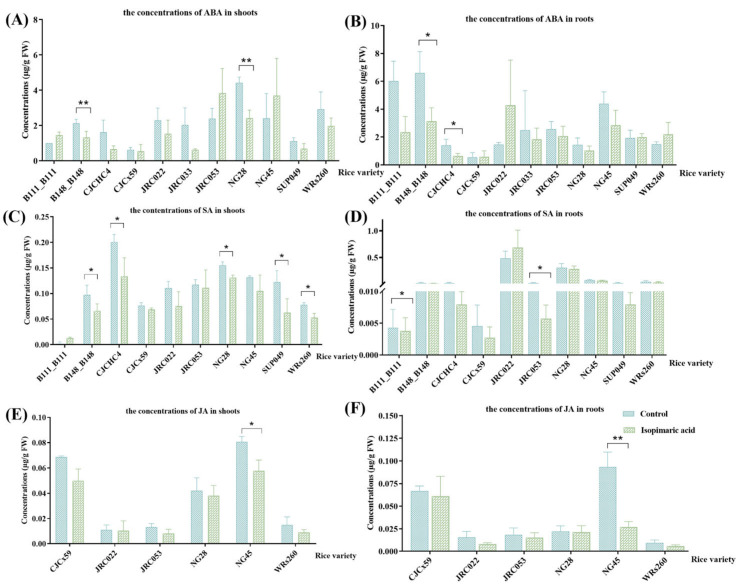
The defense-related phytohormone of 14 rice seedling varieties that received isopimaric acid. (**A**,**C**,**E**): ABA, SA, and JA concentrations in the shoots of rice seedlings treated with 25 μg/mL of isopimaric acid. (**B**,**D**,**F**): ABA, SA, and JA concentrations in the roots of rice seedlings treated with 25 μg/mL of isopimaric acid. Mean differences between two groups were compared using independent-samples *t*-tests, with *p* < 0.05 indicated by “*” and *p* < 0.01 indicated by “**”.

The concentrations of defense-related phytohormones, including abscisic acid (ABA), salicylic acid (SA), and jasmonic acid (JA), were qualitatively analyzed in the shoots and roots of rice seedlings following treatment with 25 μg/mL of isopimaric acid (Figure 5). Compared with the control group, the concentrations of ABA, SA, and JA all decreased in the shoots and roots of most tested rice varieties following treatment, with the values ranging from 0.0082 to 4.42 μg/g FW and from 0.0056 to 6.60 μg/g FW, respectively. Of these, the ABA concentrations were significantly reduced in the shoots of seedlings from two varieties, B148_B148 and NG28, with the greatest reduction in the ABA concentration (45.14 ± 0.30%) seen in the shoots of NG28 rice seedlings. 

### 3.3. Correlation Analysis of Phytohormone Concentrations with Growth Characteristics in Rice Seedlings Following Treatment with Isopimaric Acid

#### 3.3.1. Correlation Analysis of Growth-Related Phytohormone Concentrations with Growth Characteristics in Rice Seedlings

**Figure 6:**
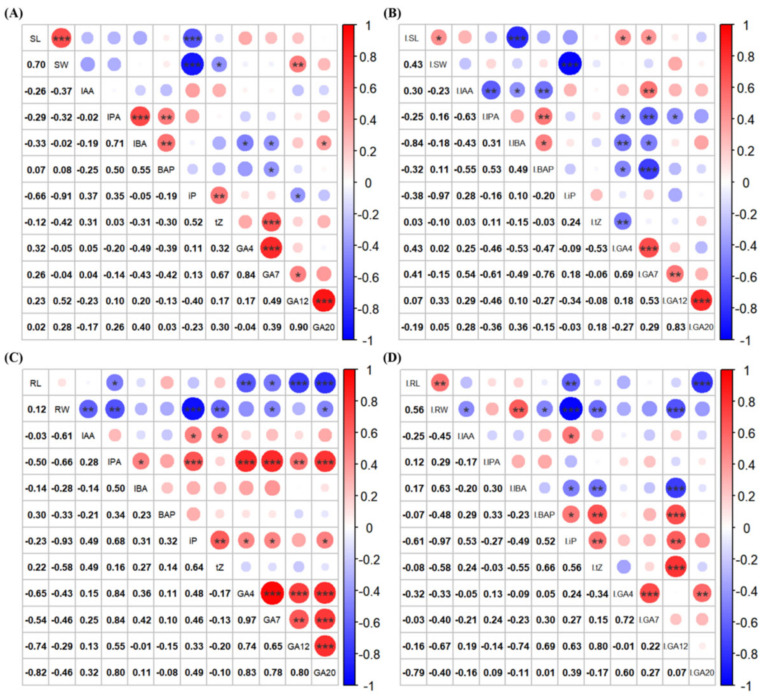
Correlation analysis of growth-related phytohormone concentrations in rice seedlings treated with isopimaric acid. (**A**): Correlation of growth-related phytohormone concentrations in the shoots of rice seedlings in the control group. (**B**): Correlation of growth-related phytohormones in the aboveground parts of rice seedlings treated with 25 μg/mL of isopimaric acid. (**C**): Correlation of growth-related phytohormone concentrations in the roots of rice in the blank control group. (**D**): Correlation of growth-related phytohormone concentrations in the roots of rice seedlings treated with 25 μg/mL of isopimaric acid. Red and blue circles represent positive and negative correlations, respectively. Mean differences between two groups were compared using independent-samples *t*-tests, with *p* < 0.05 indicated by “*”, *p* < 0.01 indicated by “**”, and *p* < 0.001 indicated by “***”. *** represents an extremely significant difference between the means of the two sets of data.

Following isopimaric acid treatment, the lengths of rice seedling shoots were positively correlated with the concentrations of four growth-related phytohormones, IAA, GA_4_, GA_7_, and GA_12_, and were found to be negatively correlated with the concentrations of IBA (*p* < 0.001). This suggests that reductions in the IBA concentration resulted in a significant increase in the shoot lengths compared with that of the control group (Figure 6). Furthermore, root weight was also positively correlated with the concentrations of two growth-related phytohormones, IPA and IBA, and highly significantly and negatively correlated with concentrations of iP (*p* < 0.001). This suggests that decreases in the concentration of iP resulted in a highly significant increase in the total root weight of rice seedlings. In addition, isopimaric acid altered the correlation between the concentrations of certain growth-related phytohormones and the lengths and weight of rice seedlings. For example, the relationship between the root weight of rice seedlings and IBA concentrations changed from a negative correlation to a significant positive correlation following treatment with isopimaric acid (*p* < 0.01).

#### 3.3.2. Correlation of Concentrations of Defense-Related Phytohormones with Growth Characteristics in Rice Seedlings

**Figure 7:**
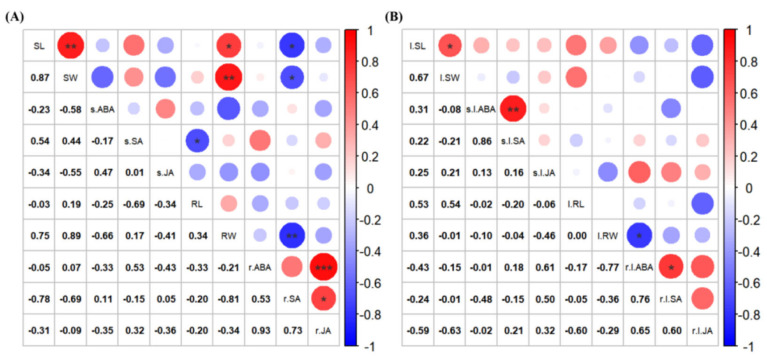
Correlation analysis of defense-related phytohormone concentrations in rice seedlings treated with isopimaric acid. (**A**): Correlation of defense-related phytohormone concentrations in rice seedlings of the control group. (**B**): Correlation of defense-related phytohormone concentrations in rice seedlings treated with 25 μg/mL of isopimaric acid. Red and blue circles represent positive and negative correlations, respectively. Mean differences between two groups were compared using independent-samples *t*-tests, with *p* < 0.05 indicated by “*”, *p* < 0.01 indicated by “**”, and *p* < 0.001 indicated by “***”. *** represents an extremely significant difference between the means of the two sets of data.

The lengths of rice seedling shoots were positively correlated with the concentration of three defense-related phytohormones, ABA, salicylic acid (SA), and JA, following treatment with isopimaric acid. However, root weight was negatively correlated with the concentrations of ABA, SA, and JA. Root weight was highly significantly and negatively correlated with the ABA concentration following treatment with isopimaric acid (*p* < 0.05), which means that the reduction in the ABA concentration resulted in a significant decrease in the root weight (Figure 7). In addition, isopimaric acid changed the correlation between some defense-related phytohormone concentrations and the length and weight of rice seedlings, with the correlation between the root weight and ABA concentration changing from negative to significantly negative (*p* < 0.05).

## 4. Discussion

### 4.1. The Diterpene Isopimaric Acid Could Be Developed as a Promising Plant-Derived Growth Regulator

Plant-derived diterpenoids are rich and diverse in structures and functions, and many have plant growth-regulating activities [21]. As such, diterpenoids are a source of discovery for new plant growth regulators. Momilactone and phytocassane both play important roles in combating environmental threats, such as pathogens that harm rice growth, including *Fusarium oxysporum* and leaf blight, as well as field weeds that compete with rice for growth space [22]. GA_4_ significantly increased the growth of *Artemisia apiacea*, significantly increasing the density of glandular hairs (*p* < 0.05) as well as increasing artemisinin production, improving the resistance of this species in complex environments [23]. Furthermore, epinodosin significantly promoted the root growth of lettuce seedlings [24].

The diterpene isopimaric acid is a highly abundant specialized metabolite in *Thuja occidentalis* L. We found that isopimaric acid significantly promoted the growth of rice seedlings. Furthermore, isopimaric acid showed higher growth-promoting activity in rice seedlings compared with gibberellin; for example, the shoot length in rice seedlings of line WRs260 showed an increase of 36.48 ± 1.66%, and the shoot weight increased by 49.00 ± 26.68% following isopimaric acid treatment. These data suggest that isopimaric acid has promise for development as a diterpenoid growth regulator. Its function in regulating both external traits, including root and shoot length and weight, as well as internal physiological processes, such as phytohormone concentrations, should contribute to increased crop yields.

Because plant growth regulators are applied to crops and are, therefore, agrochemicals that can disperse into the environment, there is concern about whether these chemicals themselves or their degradation products are harmful to non-target organisms and whether they can have adverse environmental consequences. Plant-derived growth regulators are more compatible with plants, animals, and the environment than traditional chemical growth regulators. The scientific use of such growth regulators of plant origin can therefore make a significant contribution to the sustainable development of agricultural production [25].

### 4.2. Isopimaric Acid May Act through Phytohormone Pathways to Promote Rice Growth

There are many mechanisms by which different compounds can promote plant growth, with hormones being an important pathway. For example, the sesquiterpene germacrane (1E,4E)-germacrdiene-6β,15-diol secreted by the roots of *Ambrosia trifida* promotes plant growth by regulating IAA concentrations in the inter-root bacterium *Enterobacter* sp. L LHD-19 [26]. Similarly, the diterpene precursor of kauralexins, ent-copalyl pyrophosphate, maintains plant growth by participating in the synthesis of phytogeranin [27]. Certain transcription factors controlling the expression of terpene synthases have also been shown to promote plant growth through hormonal pathways. For example, the AP2/ERF transcription factor ZmEREB92 significantly represses the expression of the ethylene-signaling gene ZmEIL7 and the α-amylase gene ZmAMYa2 during the normal suckering of maize (*Zea mays* L.) seeds, increasing ethylene concentrations and starch degradation, and ensuring timely seed germination [9]. Similarly, the rice NAC transcription factor OsNAC3 directly binds to the ABA catabolism gene OsABA8ox1 and the promoter of the cell expansion gene OsEXP4, activating their expression during rice seed germination to promote cell elongation and seed germination [28].

In this study, we found that isopimaric acid had a more pronounced growth-promoting effect on rice seedlings than gibberellin, and further measurement of the phytohormone concentrations revealed that the exogenous addition of isopimaric acid altered the concentrations of growth-related phytohormones such as GA_7_ and defense-related phytohormones such as ABA. This regulated the energy allocation between growth and defense in rice seedlings by reducing the concentration of defense-related phytohormones, thus exerting growth-promoting effects. Previous studies have focused on the antimicrobial and anticancer activities of isopimaric acid [29]. Our results reveal, for the first time, that isopimaric acid is active in plant growth and elucidate the mechanism by which it promotes the growth of rice seedlings through phytohormone pathways. The phytohormone pathways are an ideal model for studying the regulation of plant growth by plant-specialized metabolites and provide a simple platform for constructing a network of specialized metabolite products and plant growth regulation [30]. Unfortunately, we were not able to find the molecular mechanism of the hormone isopimaric acid association. Subsequent studies should focus on finding the transcription factors that regulate the growth of rice seedlings following the application of isopimaric acid and further reveal the mechanism by which isopimaric acid exerts its growth-promoting activity.

## 5. Conclusions

The diterpene isopimaric acid has a wide range of applications in agriculture and is of great value in research into seed germination and seedling growth [31]. This study investigated the biological activity of isopimaric acid on rice seedlings. We found that the application of a certain concentration of isopimaric acid had a promotional effect on the growth of rice seedlings. UPLC-MS/MS analyses revealed changes in the endogenous phytohormone concentrations in the rice plants following isopimaric acid treatment. This study confirmed that isopimaric acid, a naturally active substance, improves the growth status of rice seedlings by affecting the balance of endogenous phytohormones, and provides a research basis for the use of isopimaric acid as a plant early growth regulator. Unfortunately, we have not yet found the transcription factors that play a role in regulating the growth of rice seedlings after the application of isopimaric acid. These transcription factors could further reveal the mechanism by which isopimaric acid exerts its growth-promoting activity and will be the subject of future research.

## Data Availability

The data will be available upon request.

## Supplementary Materials

The following supporting information can be downloaded at https://www.mdpi.com/article/10.3390/cimb46090580/s1. Table S1: Formula of rice Hoagland’s nutrient solution; Table S2: MRM analysis conditions for plant hormone compounds analyzed using UPLC-MS/MS; Table S3: Calibration equations for plant hormones.
