# Supplementary material for: The Diterpene Isopimaric Acid Modulates the Phytohormone Pathway to Promote Oryza sativa L. Rice Seedling Growth
Source: Curr Issues Mol Biol. 2024 Sep 2;46(9):9772–84. doi: 10.3390/cimb46090580 (PMC11430709; doi:10.3390/cimb46090580)
Supplement: Supplementary file 1 [file cimb-46-00580-s001.zip › cimb-3168682-supplementary.pdf]

Supplementary Materials

The Diterpene Isopimaric Acid Modulates the Phytohormone Pathway to Promote *Oryza sativa* L. Rice Seedling Growth

Jiaqi Huang †, Juan Hua †, Luying Peng, Liping Bai * and Shihong Luo *

Engineering Research Center of Protection and Utilization of Plant Resources, College of Bioscience and Biotechnology, Shenyang Agricultural University, Shenyang 110866, China; jiaqihuang@stu.syau.edu.cn (J.H.); huajuan@syau.edu.cn (J.H.)

* Correspondence: bailiping@syau.edu.cn (L.B.); luoshihong@syau.edu.cn (S.L.)

† These authors contributed equally to this work.

Table S1: Formula of Rice Hoagland's nutrient solution.

	Chemical Components	concentration g/L
Massive element masterbatch（100X，10 mL/L）	$Ca(NO_3)_2·4H_2O$	23.8512
	$NH_4H_2PO_4$	1.4952
	KNO_3	51.5610
$MgSO_4$ masterbatch（100X，10 mL/L）	$MgSO_4$	5.9939
Trace element masterbatch（500X，2 mL/L）	H_3BO_3	0.2993
	$MnCl_2·4H_2O$	0.2010
	$ZnSO_4·7H_2O$	0.0451
	$CuSO_4·5H_2O$	0.0262
Iron salt masterbatch（200X，5 mL/L）	Na_2-EDTA·$2H_2O$	1.6602
	$FeSO_4·7H_2O$	1.2455

Table S2: MRM analysis conditions for plant hormone compounds analyzed using UPLC-MS/MS.

No.	Compounds	Mode	MRM transition	Q1 Pre Bais (V)	CE (V)	Q3 Pre Bais (V)
1	IAA	ESI+	176.00 > 130.00	–19	–15	–22
2	IPA	ESI+	189.85 > 130.10	–20	–16	–24
3	IBA	ESI–	201.95 > 116.05	25	19	22
4	BAP	ESI+	225.95 > 91.25	–25	–25	–20
5	iP	ESI+	203.95 > 136.20	–14	–18	–27
6	*t*Z	ESI+	220.10 > 136.05	–15	–17	–20
7	GA_4	ESI–	331.05 > 213.10	22	32	21
8	GA_7	ESI–	329.20 > 223.20	23	18	23
9	GA_{12}	ESI–	331.05 > 201.05	20	45	28
10	GA_{20}	ESI–	331.20 > 287.20	22	32	21
11	ABA	ESI–	263.10 > 153.10	20	12	28
12	SA	ESI–	137.20 > 93.20	10	15	16
13	JA	ESI–	209.25 > 59.15	15	11	15

Table S3: Calibration equations of plant hormones.

No.	Compounds	Calibration curves	R^2
1	IAA	$y = (2.03 \times 10^{-5})\,x - 4.50 \times 10^{10}$	0.9993
2	IPA	$y = (1.31 \times 10^{-7})\,x + 2.577 \times 10^{4}$	0.9984
3	IBA	$y = (7.17 \times 10^{5})\,x - 9.13 \times 10^{3}$	0.9996
4	BAP	$y = (4.85 \times 10^{-6})\,x - 0.0172$	0.9985
5	iP	$y = (1.39 \times 10^{-6})\,x + 5.75 \times 10^{-3}$	0.9990
6	*t*Z	$y = (1.17 \times 10^{-7})\,x + 2.9174 \times 10^{4}$	0.9984
7	GA_4	$y = (4.99 \times 10^{-6})\,x - 0.0077$	0.9986
8	GA_7	$y = (2.35 \times 10^{-5})\,x - 0.0839$	0.9933
9	GA_{12}	$y = (2.12 \times 10^{-5})\,x - 0.0023$	0.9933
10	GA_{20}	$y = (2.01 \times 10^{5})\,x + 1.55 \times 10^{3}$	0.9984
11	ABA	$y = (8.52 \times 10^{-6})\,x + 0.0083$	0.9987
12	SA	$y = (5.77 \times 10^{-8})\,x - 0.0038$	0.9900
13	JA	$y = (2.50 \times 10^{-7})\,x - 0.0247$	0.9986
